# Antibiotic Susceptibility-Guided Concomitant Therapy Regimen with Vonoprazan, High-Dose Amoxicillin, Clarithromycin, and Metronidazole for *Helicobacter pylori* Eradication as Fourth-Line Regimen: An Interventional Study

**DOI:** 10.3390/microorganisms12102104

**Published:** 2024-10-21

**Authors:** Soichiro Sue, Takeshi Sato, Mao Matsubayashi, Hiroaki Kaneko, Kuniyasu Irie, Shin Maeda

**Affiliations:** Department of Gastroenterology, Graduate School of Medicine, Yokohama City University, Yokohama 236-0004, Japan

**Keywords:** vonoprazan, concomitant, susceptibility testing, *Helicobacter pylori*

## Abstract

This is the first registered intervention study for vonoprazan, high-dose amoxicillin, clarithromycin, and metronidazole 14-day concomitant therapy based on a susceptibility test of *Helicobacter pylori*. We conducted this study as a fourth-line rescue regimen in Japan. Methods: Twenty patients who underwent three rounds of eradication therapies (first- or second-line 7-day triple therapy consisting of amoxicillin and clarithromycin, or metronidazole- and sitafloxacin-based third-line therapy) and had failed eradication based on a urea breath test or fecal antigen test were recruited. All patients underwent endoscopic examination and culture tests before starting eradication therapy. The intervention was concomitant therapy consisting of vonoprazan 20 mg bid, amoxicillin 500 mg qid, clarithromycin 400 mg bid, and metronidazole 250 mg bid for 14 days, which were modified based on the susceptibility test, and the resistant drugs were removed from the regimen. Patients with negative culture results were treated with quadruple therapy. The primary outcome was the eradication rate (UMIN000025765, jRCTs 031180208). Results: The eradication rate of susceptibility-testing-based fourth-line eradication therapy was 63.2% (95%CI: 38.4–83.7%) in intent-to-treat analysis and 70.6% (95%CI: 44.0–89.7%) in per-protocol analysis. Thirteen patients received quadruple therapy, with eradication rates of 61.5% and 75.0%, respectively. No serious adverse events were reported. Conclusions: This vonoprazan-based concomitant therapy modified by the susceptibility test is a potential option as fourth-line eradication after first-line clarithromycin-based 7-day triple, second-line metronidazole-based 7-day triple, and third-line sitafloxacin-based 7-day triple therapy failure.

## 1. Introduction

Long-term *Helicobacter pylori* (*H. pylori*) infection is involved in the development of gastric cancer [1,2]. A consensus has been reached indicating that the eradication of *H. pylori* can contribute to the prevention of gastric cancer [3,4,5]. Therefore, it is recommended that all patients with *H. pylori* infection undergo eradication therapy [5]. The need to select eradication regimens based on geographic region is also a consensus by the results of the Kyoto Global Consensus Meeting on *H. pylori* gastritis [5] in Japan from the 2013 national health insurance coverage for the treatment for *H. pylori*-associated chronic gastritis [6].

In Japan, a 7-day triple therapy regimen using vonoprazan (VPZ), a potassium-competitive acid blocker, or a proton pump inhibitor (PPI), along with amoxicillin (AMPC) and clarithromycin (CAM), is approved as a first-line (VPZ-AMPC-CAM, PPI-AMPC-CAM) [7,8], and a 7-day VPZ- or PPI-based triple therapy with AMPC and metronidazole (MNZ) is approved as a second-line eradication regimen (VPZ-AMPC-MNZ, PPI-AMPC-MNZ) [7,8]. As a third-line regimen, triple therapy consisting of VPZ, AMPC, and sitafloxacin (STFX) (VPZ-AMPC-STFX) or VPZ, MNZ, and STFX (VPZ-MNZ-STFX) is recommended in Japan by the following third-line clinical trial evidence [4]. Murakami et al. reported an RCT showing the superiority of PPI-AMPC-STFX (7-day triple therapy consisting of lansoprazole 30 mg two times daily (bid), AMPC 750 mg bid, and STFX 100 mg bid) compared to PPI-AMPC (14-day dual therapy consisting of lansoprazole 30 mg four times daily (qid) and AMPC 500 mg qid) and PPI-AMPC-LVFX (7-day triple therapy consisting of lansoprazole 30 mg bid, AMPC 750 mg bid, and levofloxacin (LVFX) 300 mg bid) [9]. From this result, STFX is mainly used in Japan as a quinolone antibiotic for *H. pylori* eradication [10]. Furuta et al. reported an RCT showing no significant difference between PPI-AMPC-STFX (triple therapy with rabeprazole 10 mg bid/qid, AMPC 500 mg qid, and STFX 100 mg bid) for 1 week, PPI-AMPC-STFX for 2 weeks, PPI-MNZ-STFX (triple therapy consisting of rabeprazole 10 mg bid/qid, MNZ 250 mg bid, and STFX 100 mg bid) for 1 week, and PPI-MNZ-STFX for 2 weeks [11]. We reported an RCT comparing VPZ-AMPC-STFX (7-day triple therapy consisting of VPZ, AMPC, and STFX) and PPI-AMPC-STFX (7-day triple therapy consisting of PPI, AMPC, and STFX), and VPZ-AMPC-STFX showed a significantly higher eradication rate compared to PPI-AS in per-protocol (PP) analysis, but no significant eradication rate in intension-to-treat (ITT) analysis [12].

After the STFX-based third-line eradication failure in Japan, there is no RCT evidence, and only single-arm studies have been reported [13]. Generally, there are three important factors that contribute to the failure of *H. pylori* eradication. The most important factor is whether the antibiotic used is sensitive to *H. pylori* [14]. The second is whether the dosage and duration of antibiotic use are sufficient [15]. The third is whether or not the suppression of acid secretion is sufficient [8].

A 14-day concomitant therapy is recommended by Maastricht VI guidelines as the standard first-line eradication therapy, especially in the countries including Japan where bismuth use is difficult [3]. A large-scale randomized controlled trial reported that 10-day concomitant therapy achieved a 90% eradication rate in CAM- and MNZ-susceptible subjects on an ITT basis (*n* = 211), but a longer treatment length suggested [16]. A recent meta-analysis showed a similar efficacy of concomitant therapy consisting of PPI, AMPC, CAM, and MNZ (PPI-AMPC-CAM-MNZ) compared to bismuth containing quadruple therapy (BQT) [17]. A subgroup analysis between 14-day PPI-AMPC-CAM-MNZ and 14-day BQT also showed a similar efficacy.

A recent systematic review and meta-analysis (SRM) showed that tailored therapy in the first-line setting was more effective than empirical therapy, whereas after the second-line setting, a significant difference in tailored therapy compared to empirical therapy was not observed [18]. However, a meta-analysis showed that MNZ resistance reduced the eradication rate by 37.7% with the MNZ-containing regimen, and CAM resistance reduced the eradication rate by 55% with the CAM-containing regimen [14]. For this reason, it is a general rule to treat *H. pylori* with sensitive antibiotics in the treatment of infectious diseases, including *H. pylori* eradication [19]. Thus, we planned a culture-susceptibility-test-based salvage eradication therapy, in which resistant antibiotics were not used.

For the dosage of AMPC, we planned 500 mg qid (2000 mg/day) (high-dose AMPC), more than the generally used dosage of 750 mg bid (1500 mg/day) in Japan [7]. In addition, we planned a VPZ-based regimen instead of a PPI-based one. This is based on previous evidence comparing VPZ-based versus PPI-based regimens, as we reviewed previously [8], and AMPC four times daily to reach and maintain the ideal concentration is reasonable [20], because AMPC is a time-dependent antibiotic, as we reported previously [21]. A recent SRM of an RCT for VPZ-based versus PPI-based *H. pylori* eradication also supports this setting, which showed a higher eradication rate with the 14-day VPZ-based bismuth-containing quadruple therapy (91.8%) compared to VPZ-AMPC-CAM (89.1%) or VPZ-AMPC (82.8%). This study also showed that VPZ-AMPC was as effective as VAC and superior to PPI-based therapies [22]. For the duration, a 14-day duration was proven most effective compared with shorter durations by an SRM [15]; thus, we used a duration of 14 days.

Consequently, we performed an intervention study of a susceptible-test-based modified rescue regimen with 14-day VPZ, high-dose AMPC, CAM, and MNZ as a fourth-line or later rescue eradication.

## 2. Methods

### 2.1. Study Design and Ethical Issues

This study was designed as a single-center, open-label, single-arm intervention study for susceptibility-test-based VPZ, high-dose AMPC, CAM, and MNZ 14-day concomitant therapy.

This research received ethical approval from Yokohama City University Hospital’s review board on 16 December 2016 (number B16 201006). Following the implementation of the Clinical Trials Act in 2019, this study underwent a mandatory re-evaluation and gained approval from Yokohama City University’s Institutional Review Board (CRB18-026). This research adhered to the Declaration of Helsinki and Japan’s Ethical Guidelines for Medical Research (2017). It also complied with the country’s Clinical Trials Act and was registered in both the UMIN trial registry (UMIN000025765) and the jRCTs registry (jRCTs0311802083) established in 2019. Both registries are recognized by the International Committee of Medical Journal Editors. Informed consent was obtained in writing from all participants before they were included in this study.

### 2.2. Participants

The inclusion criteria for this study were male and female adult patients (aged > 20 years); patients with *H. pylori* infection that failed first-, second- (VPZ or PPI-AMPC-CAM, VPZ or PPI-AMPC-MNZ), and STFX (sitafloxacin)-based third-line eradication; patients who were diagnosed with *H. pylori* infection; a urea breath test [23] or *H. pylori* stool antigen test was used for diagnosis [24]; and patients who could perform a urea breath test after 8 weeks from treatment completion. Both methods are standard methods for diagnosing *H. pylori* infection [4].

Patients were excluded from this study if they had any of the following criteria: past history of allergy or side-effects requiring treatment for the drugs used in this study; a patient who did not agree to endoscopy and susceptibility testing from their own medical care expenses before eradication therapy; pregnancy or lactation; patients with infectious mononucleosis; patients with brain and spinal cord disease; patients with severe renal dysfunction, severe heart dysfunction, or severe liver dysfunction; and patients who were disqualified from this study by physicians. Before participating, all participants provided written informed consent.

### 2.3. Procedure

This study was designed to conduct upper gastrointestinal endoscopy and *H. pylori* culture and susceptibility testing (detail: Section 2.4) after study enrollment. However, if endoscopy and susceptible testing had been performed immediately before study enrollment, it was not permitted to perform it again, and a positive culture result was used as a diagnosis of infection, because culture testing is the most reliable method for diagnosing *H. pylori* [25]. The eradication treatment regimen was determined based on the susceptibility testing result including a negative culture result (detail: Section 2.5). After the eradication, a urea breath test (UBT) was used at least 8 weeks after the end of eradication (detail: Section 2.6).

### 2.4. Susceptibility of H. pylori to Antimicrobial Agents

The susceptibilities of *H. pylori* to various antibiotics were determined using culture and agar dilution methods. Stomach tissue samples were taken during endoscopy and placed in a special medium for transporting *H. pylori* bacteria. These samples were then cultured to grow the bacteria. A test called an agar dilution test was used to determine the effectiveness of certain antibiotics against the bacteria. The tests were performed by a lab that did not know anything about the patients or the research. The bacteria were grown in a special type of blood agar with added carbon dioxide. They were identified based on their appearance and reactions to specific tests. If *H. pylori* was found, the effectiveness of four antibiotics, AMPC, CAM, MNZ, and STFX, was measured using a specific method: agar dilution method. The antibiotics were tested at different concentrations to see how much was needed to stop the bacteria from growing (the minimum inhibitory concentrations (MICs) of AMPC, CAM, MNZ, and STFX). The above method is the same as our previous reports [26,27]. The breakpoints for resistance to these antibiotics were defined as ≥0.5 mg/L for AMPC, ≥1.0 mg/L for CAM, ≥8 mg/L for MNZ, and ≥0.12 mg/L for STFX, based on previous studies [12,16,28].

### 2.5. Treatment

Patients who were eligible and signed the informed consent document were part of this study. The patients were assigned to receive a modified regimen based on *H. pylori* antibiotics resistance information. A 14-day concomitant therapy consisting of VPZ (vonoprazan) 20 mg bid, AMPC (amoxicillin) 500 mg qid (high-dose AMPC), CAM (clarithromycin) 400 mg bid, and MNZ (metronidazole) 250 mg bid was settled as the regimen of this study in the without-modification case. This regimen was used with modification or without modification based on susceptibility testing of *H. pylori* for AMPC, CAM, and MNZ. (1) All susceptible cases: no modification. (2) One AMPC, CAM, or MNZ is resistant: the resistant drug is removed from the regimen. (3) Two AMPC, CAM, or MNZ are resistant: the resistant drugs are removed from the regimen. (4) No susceptibility testing results: no modification. In the case with all antibiotics resistant, no intervention is conducted.

All of the patients were prohibited from taking PPI and antibiotics except for the assigned regimen in the treatment period.

Given that the primary outcome of eradication rate is objectively measurable, an open-label design was chosen for this trial.

### 2.6. Outcome

In the current study, we exploratorily checked the efficacy and safety of susceptibility-testing-based VPZ, high-dose AMPC, CAM, and MNZ 14-day concomitant therapy (14 days-VhACM).

The primary endpoint of this study was the eradication success rate of *H. pylori* as fourth-line eradication, after failure of the first-, second- (VPZ or PPI-AMPC-CAM, VPZ or PPI-AMPC-MNZ), and STFX (sitafloxacin)-based third-line eradication. Success was assessed with the urea breath test (UBT) measured at least 8 weeks after the end of the therapy. Eradication success was defined as a UBT value less than 2.5‰. Urea breath tests (UBTs) were conducted using UBIT (100 mg) tablets from Otsuka Pharmaceutical, Tokyo, Japan. UBT samples were collected at Yokohama City University Hospital by independent hospital staff, and the tests were analyzed by an external clinical inspection agency with blinded operators. All patients were required to discontinue VPZ, PPI, histamine-2 blockers, and antibiotics for at least four weeks prior to undergoing the UBT.

We conducted both intention-to-treat (ITT) and per-protocol (PP) analyses. All patients who started the treatment were included in the ITT analysis, even if they did not finish the treatment. Patients who did not undergo UBT after eradication or were lost to follow-up were considered eradication failures in the ITT analysis.

### 2.7. Safety

Safety was assessed as a secondary endpoint using a patient-completed adverse effects questionnaire (AEQ), which was consistent with our previous research. [12,26,29] The AEQ included questions about fatigue, vomiting, eructation, abdominal fullness, headache, urticaria, heartburn, abdominal pain, anorexia, nausea, dysgeusia, diarrhea, and other symptoms. Responses ranged from none (AEQ 0) to strong (AEQ 3). The AEQs were administered at the beginning of each clinical examination, ensuring that there was no reporting bias.

### 2.8. Sample Size Calculation

We set the sample size to 30 to exploratorily assess the regimen, based on the feasibility from our STFX (sitafloxacin)-based third-line eradication achievements [12] and 5-year study period. We calculated this sample size of 30 based on an 80% eradication rate of third-line eradications and based on the 30 introduction patients for third-line eradications per year.

### 2.9. Statistical Analyses

Eradication rates were calculated with 95% CI. Eradication rates were calculated for not only total-susceptibility-testing-based VPZ, AMPC, CAM, and MNZ 14-day concomitant therapy, but also for each modified regimen.

## 3. Results

### 3.1. Recruitment and Follow-Up Periods

**Figure 1:**
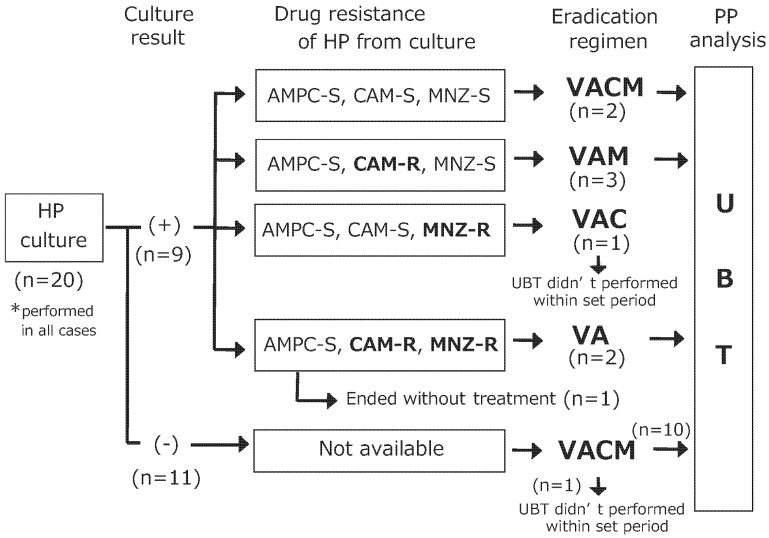
Flow chart of regimen modification based on *H. pylori* antibiotic resistance. HP, *H. pylori*; VACM, 14-day concomitant therapy consisting of vonoprazan, amoxicillin, clarithromycin, and metronidazole; VAM, 14-day triple therapy consisting of vonoprazan, amoxicillin, and metronidazole; VA, 14-day dual therapy consisting of vonoprazan and amoxicillin; VAC, 14-day triple therapy consisting of vonoprazan, amoxicillin, and clarithromycin.

This study was conducted from January 2017 to November 2021. The first patient was included in July 2017. The last patient was included in November 2020. A total of 20 cases were included. Recruitment to this study was stopped on 30 May 2021, because of the state of protocol. The observation period was completed on 12 April 2021. One case did not perform the trial treatment, because of the multidrug-resistant result from the culture sensitivity test. A total of 19 cases were included for intent-to-treat analysis. Two cases were excluded from the per-protocol analysis, because UBT was not performed within the set period. A total of 17 cases were included for per-protocol analysis as shown in Figure 1.

### 3.2. Baseline Characteristics

The baseline characteristics of *H. pylori*-infected patients who were eradicated with susceptibility-testing-based VPZ, high-dose AMPC, CAM, and MNZ 14-day concomitant therapy (14 days-VhACM) are summarized in Table 1. In eight cases, AMPC, CAM, MNZ, and STFX resistance information were collected from the *H. pylori* culture and agar plate dilution method result before the eradication. Table 1 shows an overview of the antibiotic resistance background of *H. pylori*. This group had failed eradication therapy including AMPC, CAM, MNZ, and STFX, although the resistance rates to CAM, MNZ, and STFX were high but not 100%. Furthermore, no AMPC resistance was observed despite having undergone eradication therapy including AMPC three times. Dual resistance to CAM and MNZ was observed in 50% of cases.

### 3.3. Antibiotic Susceptibility-Guided Concomitant Therapy Regimen

As shown in Figure 1, eradication regimen was modified based on *H. pylori* culture and antibiotic susceptibility testing.

### 3.4. Efficacy

The primary endpoint, eradication rate of susceptibility-testing-based fourth-line *H. pylori* eradication therapy, was 63.2% (95%CI: 38.4–83.7%) in ITT and 70.6% (95%CI: 44.0–89.7%) in PP analysis, as shown in Table 1. The regimens determined by protocol and susceptibility testing and the eradication rates are shown below. As shown in Table 2, a total of 13 in 19 cases were treated with 2 weeks of quadruple therapy consisting of VPZ, AMPC, CAM, and MNZ, and the eradication rate was 69.2% (*n* = 13) in ITT analysis and 75.0% (*n* = 12) in PP analysis. A total of 3 in 19 cases were treated with 2 weeks of triple therapy consisting of VPZ, AMPC, and MNZ, and the eradication rate was 66.7% (*n* = 3) in both ITT and PP analysis. A total of 2 in 19 cases were treated with 2 weeks of dual therapy consisting of VPZ and AMPC, and the eradication rate was 50.0% (*n* = 2) in both ITT and PP analysis. A total of 1 in 19 cases was treated with 2 weeks of triple therapy consisting of VPZ, AMPC, and CAM; UBT did not perform in the set period—0% (*n* = 1) in ITT analysis—so it was excluded from PP analysis.

Table 3 shows details of the eradication rate by a previous eradication regimen and the regimen selected for fourth-line eradication. In the subgroup in which VPZ was used in the first, second, and third lines of eradication, VACM treatment was performed, and the eradication rate was 100% (*n* = 3) in PP. There was no trend toward a higher eradication rate in the subgroup, with no history of VPZ use using a regimen containing VPZ for fourth-line eradication.

Table 4 shows the eradication rate between the with-culture result (*n* = 8) and without-culture result (*n* = 11). The with-culture-result group showed similar eradication rates to the without-culture-result group (62.5% versus 63.6% in ITT, and 71.4% versus 70% in PP analysis), despite including cases in which antibiotic use was reduced. VACM treatment for AMPC-susceptible, CAM-susceptible, and MNZ-susceptible (ALL-S) in the “with-culture-result” group achieved a 100% eradication rate (*n* = 2) in ITT and PP analysis, whereas VACM treatment in the “without-culture-result” group achieved a 63.6% eradication rate in ITT analysis (*n* = 11) and 70% in PP analysis (*n* = 10).

### 3.5. Adverse Events

We assessed patients who completed the adverse event questionnaire (AEQ), as shown in Table 5. All cases including AEQ3 and AEQ2 were finally assessed as CTCAE grade1 adverse events by a physician. The were 12 CTCAE grade1 adverse events in 8 cases (40%, 8/20): 5 events of diarrhea, 2 events of abdominal fullness, 2 events of heartburn, 1 event of abdominal pain, 1 event of dysgeusia, and 1 event of belching. All adverse events were spontaneously cured without intervention. No patients were hospitalized because of adverse events. This score is meaningful for comparing to our previous studies.

## 4. Discussion

As far as we know, this is the first intervention study to evaluate the efficacy and safety of susceptibility-testing-based VPZ, high-dose AMPC, CAM, and MNZ 14-day concomitant therapy (14 days-VhACM) for patients with *H. pylori* infection that failed the first-, second- (7-day triple therapy consisting of PPI or VPZ, AMPC, and CAM, and one week of triple therapy consisting of PPI or VPZ, AMPC, and MNZ) and STFX (sitafloxacin)-based third-line eradication [22]. In addition, this study is not only the first report on VPZ-based concomitant therapy based on susceptibility testing, but also the first report on VPZ-based concomitant therapy using high-dose AMPC for 14 days [22]. VPZ-based concomitant therapy consisting of AMPC, CAM, and MNZ has not been reported to date [30]. This study showed a 63.2% eradication rate in ITT and 70.6% eradication rate in PPS analysis. This study also showed safety: all adverse events were spontaneously cured without intervention and with no hospitalization.

In this study, *H. pylori* culture susceptibility testing was performed before treatment, which revealed the distribution of *H. pylori* resistance status after first-line and second-line eradication under insurance treatment, as well as third-line eradication with STFX-based treatment. The resistance to AMPC was 0%, that to CAM was 75%, that to MNZ was 50%, and that to STFX was 87.5%. In other words, it was confirmed that AMPC was unlikely to develop resistance even in patients with a history of treatment with AMPC. On the other hand, the resistance rates for the other drugs were highest for STFX, CAM, and MNZ, but not all patients developed resistance. In recent years, attention has been focused on the problem of the inappropriate use of antibiotics, such as the use of clarithromycin for clarithromycin-resistant bacteria [31]. The fact that a certain level of effectiveness can be demonstrated by optimizing the dose and duration, even in cases where antibiotics have been used previously, is believed to be a significant result from the perspective of making effective use of antibiotics with limited susceptibility. The results of this study suggest that in first-line and second-line eradication, the eradication rate can be expected to be improved by extending the VPZ-based therapy from 7 days to 14 days and changing the dosage of AMPC to 2000 mg four times, which is rational based on the mechanism.

As we reviewed previously, numerous reports have been published regarding the efficacy of VPZ-based eradication therapy, particularly for first-line eradication, second-line eradication, and STFX-based third-line eradication [8]. Many RCTs comparing VPZ-based eradication therapies have been conducted, and SRMs have been reported; however, no reports have been published on VPZ-based concomitant therapy [22]. The aforementioned SRM reported that VPZ-based 14-day bismuth quadruple therapy had a higher eradication rate than other VPZ-based therapies and PPI-based therapies (VPZ-AMPC-CAM, VPZ-AMPC, PPI-AMPC-CAM). In general, in situations where bismuth cannot be used, concomitants are positioned as the “best available” first-line eradication option [3]. Therefore, in areas where the use of bismuth is difficult, including Japan, VPZ-based concomitant therapy is considered a promising treatment candidate.

As for the mechanism of VPZ containing this regimen, VPZ inhibits H+/K+ ATPases in a rapid, strong, and stable manner compared to other PPIs, which enable *H. pylori* to enter the growth phase at pH > 5 [7,32]. *H. pylori* is more susceptible to antibiotics in the growth phase, especially AMPC and CAM [33]. In fact, the results of a meta-analysis comparing VPZ, AMPC, and CAM with PPI, AMPC, and CAM in first-line eradication therapy [34,35], as well as a meta-analysis comparing VPZ, AMPC, and MNZ with PPI, AMPC, and MNZ in second-line eradication therapy, have been reported [36], and in both situations, they showed that the eradication rate was greater when VPZ was used.

This study suggests the possibility of using susceptibility testing based on VPZ, AMPC, CAM, and MNZ 14-day concomitant therapy outside of the fourth-line eradication regimen. In another study, we reported that the resistance rate of STFX, which is the standard drug used in Japan, was 37.9% in a group that had no history of use in eradication therapy [27]. Therefore, it is necessary to perform culture susceptibility testing as in this study and select a regimen using a sensitive drug after the third-line eradication regimen in Japan [11,12]. In cases of STFX resistance, the susceptibility testing based on VPZ, AMPC, CAM, and MNZ 14-day concomitant therapy investigated in this study is considered a candidate for third-line eradication therapy. Furthermore, it is suggested that it could be used as a first-line eradication therapy outside of Japan, where there are no restrictions on health insurance coverage [16].

This study showed the safety of the regimen, because all adverse events were classified as CTCAE grade1—not serious and resolved rapidly. These safety conclusions are consistent with previous findings from concomitant therapy [16,37].

As shown in Table 4, this study included subjects who had previously undergone PPI-based first-, second-, and third-line eradication, but it is expected that the combination of first-line VPZ-AMPC-CAM eradication, second-line VPZ-AMPC-MNZ eradication, and third-line VPZ-AMPC-STFX eradication will become more common in the future. In such cases, further studies will be necessary to determine the degree of eradication rate that this treatment can achieve in the fourth line of eradication.

As for the possible mechanisms of resistance to treatments, important factors in the success of eradication regimens include (1) the use of sensitive antibiotics, (2) sufficient acid secretion suppression, (3) a sufficient treatment period, and (4) an appropriate dosage and administration of antibiotics. Regarding (2), the use of vonoprazan is expected to suppress acid secretion more sufficiently than conventional PPIs. The treatment period for (3) was 14 days, which is assumed to be sufficient. Regarding (4), the dosage and administration of amoxicillin, 500 mg four times a day, are expected to be more effective than the usual dosage of 750 mg twice a day in Japan, but for other drugs, the dosage and administration approved for eradication treatment in Japan were adopted, and there may be room for improvement. Regarding (1), attention must be paid to the limitations of culture sensitivity tests, and there is a possibility that the test will show sensitivity even if a very small proportion of resistant bacteria are included. This study addressed the important factors (1) to (4) for the success of eradication as much as possible, but it is believed that the failure was due to one or a combination of factors being insufficient for successful eradication.

The present study had several limitations. First, this study was conducted as a single-arm intervention study in the special subject after the first-, second-, and STFX-based third-line failure in Japan, so there are limitations to generalizing the results to other populations. Second, we did not enroll the feasibility-based sample size of 30; we enrolled 20 in the 5-year study period approved by the Certified Institutional Review Board. As a result, the 95% confidence interval is wider than originally expected, and further study is expected to evaluate this susceptibility-testing-based fourth-line therapy.

In recent years, eradication therapy using rifabutin has been attracting attention as a salvage eradication therapy [38], but this study is significant in that it shows the possibility of effectively utilizing antibiotics used in health insurance medical care in Japan, such as AMPC, CAM, and MNZ, even if the antibiotic has a history of use. As in this study, in salvage treatment, it is essential to check drug susceptibility and use antibiotics that are sensitive, and this study showed that antibiotics can be reused even if they have been used in the past, as long as they are sensitive. Actually, the “with-culture-result” group showed similar eradication rates to the “without-culture-result” group, despite including cases in which antibiotic use was reduced as shown in Table 5. There are only a limited number of antibiotics that can be used in the salvage treatment of *H. pylori*, and in Japan, AMPC, CAM, MNZ, STFX (sitafloxacin), and RBT (rifabutin) appear to be the main options. Salvage eradication treatment using STFX or RBT is important [12,13], even in cases where the bacteria are resistant to AMPC, CAM, or MNZ, but on the other hand, rather than using STFX or RBT lightly, salvage eradication treatment using these more-proven antibiotics for a sufficient period of time should be considered in cases where the bacteria are sensitive to AMPC, CAM, or MNZ.

## 5. Conclusions

This study showed the potential of susceptibility-testing-based VPZ, high-dose AMPC, CAM, and MNZ 14-day concomitant therapy (14 days-VhACM) as fourth-line eradication in Japanese situation. Further study is necessary to generalize this study’s results.

## Figures and Tables

**Table 1:** Patient characteristics and *Helicobacter pylori* eradication rates.

Characteristics	Total (*n* = 20)
Age, mean ± SE, range	64 ± 11 (45–87)
Male sex, n (%)	10/20 (50%)
Cigarette smoking, n (%)	3/20 (15%)
Endoscopic findings	
Gastritis only, n (%)	20/20 (100%)
Diagnosis methods of *H. pylori* infection **^a^**	
UBT, n (%)	16/20 (80.0%)
*H. pylori* stool antigen test ^b^, n (%)	3/20 (15.0%)
*H. pylori* culture, n (%)	1/20 (5.0%)
Drug resistance information available from culture, n (%)	8/20 (40.0%)
AMPC resistance, n (%)	0/8 (0.0%)
CAM resistance, n (%)	6/8 (75%)
MNZ resistance, n (%)	4/8 (50.0%)
STFX resistance, n (%)	7/8 (87.5%)
CAM and MNZ resistance, n (%)	
CAM-susceptible and MNZ-susceptible	2/8 (25%)
CAM-susceptible and MNZ-resistant	0/8 (0%)
CAM-resistant and MNZ-susceptible	2/8 (25%)
CAM-resistant and MNZ-resistant	4/8 (50%)
Eradication conducted, %	95% (19/20)
Eradication evaluation by UBT, %	100%
Eradication rate, % (90% CI) (ITT)	63.2% (38.4–83.7%), *n* = 19
Eradication rate, % (90% CI) (PP)	70.6% (44.0–89.7%), *n* = 17

SE, standard error; Evaluation by UBT, eradication success rate determined by the ^13^C-urea breath test; UBT, ^13^C-urea breath test; eradication evaluation by UBT, %; eradication success rate deter-mined by the 13C-urea breath test; diagnosis of *H. pylori* infection, %; diagnosis method of *H. pylori* infection before eradication therapy; AMPC, amoxicillin; CAM, clarithromycin; MNZ, metronidazole; STFX, sitafloxacin; CI, confidence interval; ITT, intention-to-treat analysis; PP, per-protocol analysis. ^a^ Diagnosis method for *H. pylori* infection before eradication therapy. ^b^ Eradication success rate determined by the *H. pylori* stool antigen test.

**Table 2:** Efficacy according to individual regimens.

Regimen	VACM	VAM	VA	VAC
*n*	13	3	2	1
Eradication rate, % (95%CI) (ITT)	69.2% (38.6–90.9%)	66.7% (9.4–99.2%)	50.0% (1.3–98.7%)	0% (0.0–97.5%)
Eradication rate, % (95%CI) (PP)	75% (42.8–94.5%)	66.7% (9.4–99.2%)	50.0% (1.3–98.7%)	NA

VACM, 14-day concomitant therapy consisting of vonoprazan, amoxicillin, clarithromycin, and metronidazole; VAM, 14-day triple therapy consisting of vonoprazan, amoxicillin, and metronidazole; VA, 14-day dual therapy consisting of vonoprazan and amoxicillin; VAC, 14-day triple therapy consisting of vonoprazan, amoxicillin, and clarithromycin; CI, confidence interval; ITT, intention-to-treat analysis; PP, per-protocol analysis; NA, not available.

**Table 3:** Eradication rates with eradication regimen and history.

Eradication History			Regimen	Eradication Rate	
1st	2nd	3rd	4th (This Study)	ITT	PP
PPI-AC	PPI-AM	VAS	14 days-VACM	66.7% (2/3)	66.7% (2/3)
PPI-AC	PPI-AM	VAS	14 days-VAM	50% (1/2)	50% (1/2)
PPI-AC	PPI-AM	VAS	14 days-VAC	0% (0/1)	NA
PPI-AC	PPI-AM	PPI-AS	14 days-VACM	60% (3/5)	60% (3/5)
PPI-AC	PPI-AM	PPI-AS	14 days-VAM	100% (1/1)	100% (1/1)
PPI-AC	PPI-AM	PPI-AS	14 days-VA	50% (1/2)	50% (1/2)
VAC	VAM	VAS	14 days-VACM	75% (3/4)	100% (3/3)
VAC	VAM	PPI-AS	14 days-VACM	100% (1/1)	100% (1/1)

1st, first-line eradication of *H. pylori*; 2nd, second-line eradication of *H. pylori*; 3rd, third-line eradication of *H. pylori*; 4th, fourth-line eradication of *H. pylori*; PPI-AC, 7-day triple therapy consisting of PPI, amoxicillin, and clarithromycin; PPI-AM, 7-day triple therapy consisting of PPI, amoxicillin, and metronidazole; PPI-AS, 7-day triple therapy consisting of PPI, amoxicillin, and sitafloxacin; VAC, 7-day triple therapy consisting of vonoprazan, amoxicillin, and clarithromycin; VAM, 7-day triple therapy consisting of vonoprazan, amoxicillin, and metronidazole; VAS, 7-day triple therapy consisting of vonoprazan, amoxicillin, and sitafloxacin; 14 days-VACM, 14-day concomitant therapy consisting of vonoprazan, amoxicillin, clarithromycin, and metronidazole; 14 days-VAM, 14-day triple therapy consisting of vonoprazan, amoxicillin, and metronidazole; 14 days-VA, 14-day dual therapy consisting of vonoprazan and amoxicillin; 14 days-VAC, 14-day triple therapy consisting of vonoprazan, amoxicillin, and clarithromycin; CI, confidence interval; ITT, intention-to-treat analysis; PP, per-protocol analysis; NA, not available.

**Table 4:** Efficacy according to with- or without-culture results.

	(a) with Culture Result	(b) without Culture Result
*n*	8	11
Used regimens	VACM (*n* = 2), VAM (*n* = 3), VAC (*n* = 1), VA (*n* = 2)	VACM (*n* = 11)
Susceptibility	ALL-S (*n* = 2), CAM-R (*n* = 3), MNZ-R (*n* = 1), CAM&MNZ-R (*n* = 1)	NA
Eradication rate	62.5%	63.6%
% (95% CI) (ITT)	(24.5–91.5%)	(30.8–89.1%)
Eradication rate	71.4%	70.0%
% (95% CI) (PP)	(29.0–96.3%)	(34.8–93.3%)

VACM, 14-day concomitant therapy consisting of vonoprazan, amoxicillin, clarithromycin, and metronidazole; VAM, 14-day triple therapy consisting of vonoprazan, amoxicillin, and metronidazole; VA, 14-day dual therapy consisting of vonoprazan and amoxicillin; VAC, 14-day triple therapy consisting of vonoprazan, amoxicillin, and clarithromycin; CI, confidence interval; ITT, intention-to-treat analysis; PP, per-protocol analysis; NA, not available; ALL-S, amoxicillin-, clarithromycin- and metronidazole-susceptible; CAM-R, clarithromycin resistance (amoxicillin- and metronidazole-susceptible); MNZ-R, metronidazole resistance (amoxicillin- and clarithromycin-susceptible); CAM&MNZ-R, clarithromycin and metronidazole resistance (amoxicillin-susceptible).

**Table 5:** Adverse effects of treatment by questionnaire (*n* = 17).

	AEQ 1, 2, or 3	AEQ 2 or 3	AEQ 3
Diarrhea	58.8%	29.4%	5.9%
Dysgeusia	23.5%	11.8%	5.9%
Nausea	23.5%	5.9%	5.9%
Anorexia	17.6%	5.9%	5.9%
Abdominal pain	52.9%	5.9%	5.9%
Heartburn	23.5%	17.6%	5.9%
Hives	11.8%	5.9%	0%
Headache	17.6%	5.9%	0%
Abdominal fullness	35.3%	17.6%	0%
Belching	41.2%	17.6%	5.9%
Vomiting	0%	0%	0%
General malaise	17.6%	5.9%	0%
Other	29.4%	0%	0%

AEQ, adverse-effect questionnaire; AEQ1, weak; AEQ2, moderate; AEQ3, strong.

## Data Availability

The data are not publicly available, due to ethical concerns, but can be obtained from the corresponding author upon request.
